# Predictors of functional capacity as measured by the Glittre activities of daily living test in women with rheumatoid arthritis

**DOI:** 10.1590/1414-431X202010040

**Published:** 2021-03-15

**Authors:** M.J.A. Palugan, A.C.B. Assis, E.J.C. Bessa, A.S. Ferreira, A.J. Lopes

**Affiliations:** 1 Programa de Pós-Graduação em Ciências da Reabilitação, Centro Universitário Augusto Motta, Rio de Janeiro, RJ, Brasil; 2 Programa de Pós-Graduação em Ciências Médicas, Faculdade de Ciências Médicas, Universidade do Estado do Rio de Janeiro, Rio de Janeiro, RJ, Brasil

**Keywords:** Rheumatoid arthritis, Functional capacity, Glittre activities of daily living test, Pulmonary function tests, Nitrogen single-breath washout test

## Abstract

Although pulmonary involvement is the most common extra-articular manifestation of rheumatoid arthritis (RA), traditional pulmonary function tests (PFTs) do not show a good correlation with the field tests usually performed in these patients. In recent decades, measurement of ventilation distribution heterogeneity through the nitrogen single-breath washout (N_2_SBW) test and evaluation of functional capacity during exercise using the Glittre activities of daily living test (GA-T) have been increasingly used. Therefore, the objective of this study was to evaluate predictors of GA-T outcomes in women with RA considering demographic, anthropometric, clinical, functional variables, and chest computed tomography (CT) findings. Forty-three women with RA underwent the GA-T, the N_2_SBW test, spirometry, measurement of the diffusing capacity for carbon monoxide (DLco), measurement of respiratory muscle strength, and evaluation of physical function of the lower and upper limbs through the Health Assessment Questionnaire Disability Index (HAQ-DI). Chest CT scans were analyzed retrospectively. The GA-T time showed significant correlations with the DLco (r_s_=-0.397, P=0.008), forced vital capacity/DLco (r_s_=0.307, P=0.044), phase III slope of the N_2_SBW test (SIIIN_2_, r_s_=0.644, P<0.0001), and the HAQ-DI (r_s_=0.482, P=0.001). Disease extent as assessed by chest CT was associated with the GA-T time. On multiple regression analysis, the SIIIN_2_ and HAQ-DI were the only predictors of the GA-T time, explaining 40% of its variability. Thus, ventilation distribution heterogeneity and worse physical function substantially explain the variability in GA-T time in women with RA and varying extents of disease on chest CT.

## Introduction

Rheumatoid arthritis (RA) is a chronic and systemic inflammatory autoimmune disease of unknown etiology (1). The disease is characterized by articular and extra-articular manifestations, affecting approximately 1% of the world's population (2). As the central pathology of RA occurs in the synovial membrane, limited joint range of motion is a typical manifestation of the disease, in which long-lasting and repeated episodes of inflammation cause permanent changes in the structure and function of the joints (3). In addition to most patients with RA also suffering from muscle dysfunction and an increased risk of pulmonary disease, this limited range of motion contributes to worsening of physical function, quality of life, and functional capacity (1,4).

Pulmonary involvement in RA is responsible for up to 20% of the overall mortality in this population (5-8). The incidence of RA-related pulmonary disease (RA-PD) varies significantly in epidemiological studies, ranging from 10-70%, depending on the definition and methodology used to detect it and on the population studied (8). This disease may present with a functional pattern of restriction and/or obstruction as a consequence of parenchymal nodules, pleural involvement, airway disease, pulmonary vasculitis, and interstitial lung disease (ILD) (5). Computed tomography (CT) and pulmonary function tests (PFTs) are the main tests for the initial evaluation and follow-up of patients with RA-PD. However, both CT and traditional PFTs, including spirometry and the diffusing capacity for carbon monoxide (DLco), lack sensitivity for ventilation distribution heterogeneity and for small airway disease (SAD) (9). Associations between the severity of RA and the presence of pulmonary involvement have been shown (7). In this context, there are still uncertainties about the association between RA-PD and functional disability and which outcome measures can best evaluate the progression of lung damage (6).

Although ILD is a well-known manifestation of RA, SAD may be the most common form of pulmonary involvement in RA (7). SAD is characterized by a progressive increase in resistance as the lung empties, regional heterogeneity of the flow and time constants, and premature closure of the airways (10). With the increasing use of the nitrogen single-breath washout (N_2_SBW) test, there has been growing interest in the evaluation of SAD and ventilation distribution in various clinical conditions, including RA (9). Although inhomogeneity in ventilation is a common finding in patients with RA (9), the power of the N_2_SBW test in predicting the consequences for the patient remains unknown. Because RA is an aggressive and disabling disease with a considerable impact on quality of life and well-being, patient-based measures are important because they measure the perspective of an individual regarding the disease burden. These measures are focused on assessing pain, overall status, and functional disability, especially during exercise (11,12). Although part of functional disability during exercise in this population may be attributed to pulmonary involvement, the correlations between PFTs and field tests with submaximal exercise levels, such as the 6-min walk test (6MWT), are not good (2,13).

Considering that RA is a multisystem disease that can diffusely involve the lungs in addition to affecting the musculoskeletal system, there is an interest in developing a test that evaluates these patients beyond walking activity. Accordingly, Skumlien et al. (14) described a field test encompassing multiple tasks mimicking activities of daily living (ADLs) that involves the function of both the lower and upper limbs [the Glittre ADL test (GA-T)]. The test reproduces daily activities such as arm activities without support, rising from a chair, walking, climbing stairs, reaching, hand gripping, and moving weight in a field test-setting (14). Thus, the GA-T may be of interest for assessing the functional exercise capacity of RA patients due to the challenges imposed by the test in these patients, including deformities of the foot and knee joints causing difficulties in walking, climbing stairs, and squatting, and deformities of the hand joints impacting tasks requiring manual skill (15,16). In addition to musculoskeletal involvement, respiratory system involvement can also represent another challenge in completing the GA-T because pulmonary damage is the most important extra-articular change in RA (7,8). Because the GA-T incorporates a set of tasks that simulate ADLs and requires oxygen consumption higher than the 6MWT (17), it is potentially an interesting tool to evaluate the impact of ventilation distribution on the performance of individuals with RA during the test. We hypothesized that in patients with RA, difficulties in completing the multiple tasks of the GA-T can be explained by a longer time since diagnosis, a greater extent of lung disease, ventilation distribution heterogeneity, and musculoskeletal involvement. Therefore, the objective of this study was to evaluate predictors of GA-T outcomes in women with RA considering demographic, anthropometric, clinical, and functional variables and chest CT findings.

## Material and Methods

### Patients

Between June and November 2019, a cross-sectional study was conducted with 52 women with RA aged ≥18 years. These patients were seen regularly at the Piquet Carneiro Polyclinic of the State University of Rio de Janeiro, Brazil. All participants met the diagnostic criteria for RA proposed by the American College of Rheumatology/European League Against Rheumatism (ACR/EULAR) 2010 (18) and were diagnosed by a rheumatologist. The following exclusion criteria were used: evidence of overlap with other rheumatic diseases; infectious respiratory exacerbations in the last four weeks; smokers or ex-smokers who smoked >10 pack-years; a history of chronic obstructive pulmonary disease (COPD), asthma, or pleuropulmonary tuberculosis; restricted movement due to skin lesions or other autoimmune disorders; presence of comorbidities unrelated to RA, such as orthopedic and neurological disorders and injuries from accidents; prior surgery on the upper limbs, hip, or lower limbs; and an inability to perform the functional exercise capacity tests.

The protocol was approved by the Research Ethics Committee of the State University of Rio de Janeiro under CAAE No. 87594518.4.0000.5259, and all patients signed an informed consent form.

### Measurements

#### Lung function

Spirometry, the measurement of DLco, and the measurement of respiratory muscle strength were performed using HD CPL equipment (nSpire Health, Inc., USA) following previous recommendations (19). The results of these tests are reported as the percentage of predicted values (20-22). In addition, the N_2_SBW test was carried out in an HDpft 3000 testing system (nSpire Health, Inc.) following established standards (23). To evaluate ventilation distribution heterogeneity, we used the phase III slope of the N_2_SBW test (SIIIN_2_) (23-26), and the results are reported as the percentage of predicted values (27).

#### Clinical Disease Activity Index (CDAI)

The CDAI is calculated from four variables as follows: number of tender joints; number of swollen joints; patient's global assessment of disease activity (visual analog scale); and physician's global assessment of disease activity (visual analog scale) (28). The CDAI can vary between 0 and 76, and a higher value indicates greater disease activity.

#### Health Assessment Questionnaire Disability Index (HAQ-DI)

The HAQ-DI was used to estimate disability based on self-reported activity limitations. The HAQ-DI is derived from a musculoskeletal-targeted questionnaire including questions related to fine movements of the upper extremities and motor activities of the lower limbs (11). This questionnaire was validated and used extensively in studies and in clinical practice and consists of 20 questions of activities, which are divided into eight domains, as follows: dressing; getting up; eating; walking; hygiene; reaching; gripping; and other usual activities (3). To quantify the values found, the HAQ-DI has a score that ranges from 0 (no disability) to 3 (maximum disability). The total score is given by the mean score of the eight categories.

#### Glittre ADL test

**Figure 1:**
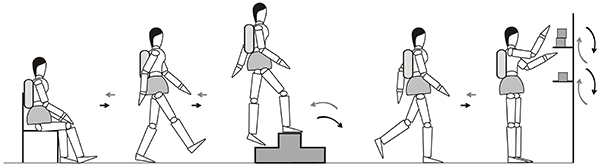
The Glittre activities of daily living test with multiple tasks.

As previously described, the GA-T (Figure 1) was performed (14). This test consisted of performing five laps in the shortest possible time in a 10-m circuit while carrying a 2.5-kg (women) backpack. In this circuit, the following activities were performed. From a sitting position, the participant walked along the course, traversing a staircase at the midpoint of the course with two ascending steps and two descending steps (17 cm high × 27 cm long). After traversing the remainder of the circuit, the participant encountered a shelf containing 31-kg objects placed on the highest shelf, and had to move the objects one by one to the lowest shelf and then to the floor. Then, the objects needed to be moved back to the lowest shelf and then placed on the highest shelf. The participant then walked back the route that she came; immediately after, another lap was started, with the exact same circuit. The protocol was performed twice, at an interval of 30 min, and the shortest GA-T time was used for analysis (29,30).

#### Chest CT

We evaluated chest CT tests that were performed within the last 3 months prior to recruitment. According to the extent of involvement of the lung parenchyma, CT scans were categorized as limited (<20%) or extensive (>20%) (31).

### Statistical analysis

Data analysis was performed using SAS 6.11 software (SAS Institute, Inc., USA). The results are reported as medians and interquartile ranges or as frequencies (percentages). Nonparametric methods were applied because the response variable “GA-T time” did not have a normal distribution (Gaussian), as the assumption of normality was rejected by the Shapiro-Wilk test. The associations of GA-T time with demographic, anthropometric, and clinical data, pulmonary function parameters, and physical function data were analyzed using Spearman's correlation coefficient (r_s_), while the association between GA-T time and CT results was analyzed using the Mann-Whitney test. Stepwise forward regression analysis was applied to identify the independent demographic, anthropometric, clinical, and functional variables and chest CT findings that explain the GA-T time. Multicollinearity was assessed using the variance inflation factor (VIF); a VIF value >5 indicates that the associated regression coefficients are poorly estimated (32). Due to the lack of a normal distribution for GA-T time, logarithmic transformation (natural log) was performed to better fit the regression model. Intraobserver agreement was evaluated using the intraclass correlation coefficient (ICC) with a two-way random model and absolute agreement and by Bland-Altman graphical analysis. P<0.05 was considered statistically significant.

## Results

The evaluated sample consisted of 43 women with RA; of the original sample, nine were excluded for the following reasons: history of smoking >10 pack-years (n=4); diagnosis of COPD (n=2); difficulty walking (n=2); and report of hip surgery (n=1). The median age was 57 (48-66) years, while the median time since diagnosis was 14 (7-18) years. Regarding the use of drugs for RA, 32, 16, and 5 reported using methotrexate, leflunomide, and prednisone, respectively. Twenty-seven participants had a CDAI>10, while 10 had a HAQ-DI≥2. Regarding lung function, 12 participants had forced vital capacity (FVC) <80% of predicted, 19 had DLco <80% of predicted, and 25 had SIIIN_2_> 120% of predicted. On CT, 33 and 10 patients had limited and extensive involvement, respectively, and no patient had a normal CT result. Regarding GA-T, the median (interquartile range) time to perform the tasks was 360 (240-540) s, which was 115% higher than the time expected to complete it using predicted values for healthy Brazilian women (30). The clinical, pulmonary function, physical function, and functional capacity data are provided in Table 1.

**Table 1:** Clinical data, lung function, physical function, and functional capacity of women with rheumatoid arthritis.

Variables	Data
Clinical data	
Age (years)	57 (48-66)
Weight (kg)	64 (57.8-75.7)
Height (cm)	156 (151-163)
BMI (kg/m^2^)	27.6 (23.7-30.1)
Time since diagnosis (years)	14 (7-18)
CDAI (score)	13 (8-18)
Lung function	
FVC (% predicted)	95 (82-104)
DLco (% predicted)	85 (69-95)
FVC/DLco (%)	1.11 (0.99-1.26)
MIP (% predicted)	70 (63-100)
MEP (% predicted)	59 (39-72)
SIIIN_2_ (% predicted)	136 (85-314)
Physical function	
HAQ-DI (points)	1.05 (0.65-1.62)
Functional capacity	
Glittre-ADL test time (s)	360 (240-540)

Data are reported as medians and interquartile ranges. BMI: body mass index; CDAI: Clinical Disease Activity Index; FVC: forced vital capacity; DLco: diffusing capacity for carbon monoxide; MIP: maximal inspiratory pressure; MEP: maximal expiratory pressure; SIIIN_2_: phase III slope of the nitrogen single-breath washout; HAQ-DI: Health Assessment Questionnaire Disability Index; ADL: activities of daily living.

**Figure 2:**
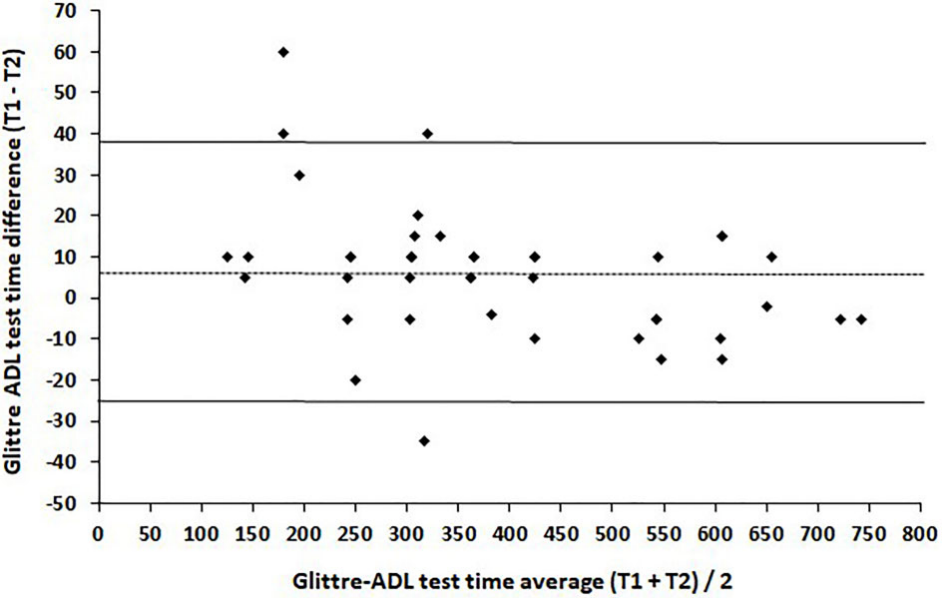
Bland-Altman plot for the total time between the 2 tests (T1 and T2).

When comparing the medians (interquartile ranges) of the 2 GA-T trials performed by the participants, no significant difference was noted, although the time to complete the second trial was shorter [365 (260-545) s *vs* 360 (250-540) s, P=0.81]; highly significant intraobserver agreement (P<0.0001) was observed between the measurements of the 2 GA-T trials (ICC=0.97 and 95%CI: 0.96-0.99). Moreover, based on the Bland-Altman plot, only 4 points outside the agreement interval and a random distribution of differences along the respective mean values (no bias) were observed, thus showing good agreement for the GA-T time between the 2 GA-T trials for women with RA (Figure 2).

**Figure 3:**
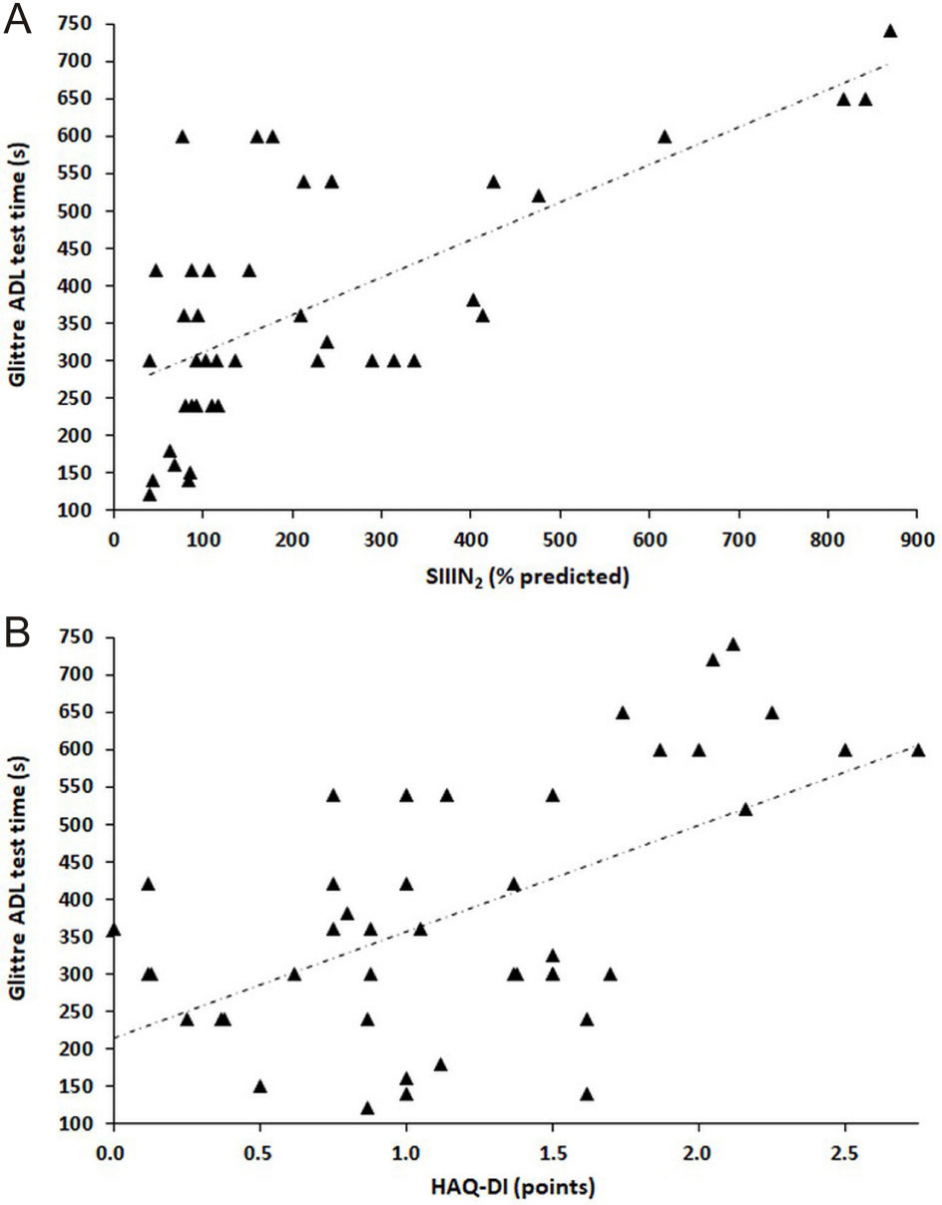
A, Relationship of Glittre activities of daily living (ADLs) test time with the phase III slope of the nitrogen single-breath washout test (SIIIN_2_, r_s_=0.644, P<0.0001) and **B**, with the Health Assessment Questionnaire Disability Index (HAQ-DI, r_s_=0.482, P=0.001). Spearman’s correlation test was used for statistical analyses.

In addition, we investigated the associations between the time to complete the multiple tasks of the GA-T and demographic, anthropometric, clinical, and functional variables (Table 2 and Figure 3). The time to perform the GA-T multitasks showed significant correlations: DLco (r_s_=-0.397, P=0.008), FVC/DLco (r_s_=0.307, P=0.044), SIIIN_2_ (r_s_=0.644, P<0.0001), and HAQ-DI (r_s_=0.482, P=0.001). The median (interquartile range) time to perform the multiple tasks of the GA-T was significantly different between patients with limited and extensive involvement on CT [300 (240-420) *vs* 570 (345-668) s, respectively; P=0.002)].

**Table 2:** Spearman's correlation coefficients for the association of Glittre ADL-test time with demographic, anthropometric and clinical data, lung function, and physical function.

Variables	Glittre ADL test time (r_s_)	P value
Age (years)	0.269	0.081
Weight (kg)	0.145	0.35
Height (cm)	-0.195	0.21
BMI (kg/m^2^)	0.197	0.21
Time since diagnosis (months)	-0.007	0.97
CDAI (score)	0.281	0.067
FVC (% predicted)	-0.294	0.055
DLco (% predicted)	-0.397	**0.008**
FVC/DLco (%)	0.307	**0.044**
MIP (% predicted)	0.102	0.51
MEP (% predicted)	0.121	0.44
SIIIN_2_ (% predicted)	0.644	**<0.0001**
HAQ-DI (points)	0.482	**0.001**

Bold type indicates significant correlations. BMI: body mass index; CDAI: Clinical Disease Activity Index; FVC: forced vital capacity; DLco: diffusing capacity for carbon monoxide; MIP: maximal inspiratory pressure; MEP: maximal expiratory pressure; SIIIN_2_: phase III slope of the nitrogen single-breath washout; HAQ-DI: Health Assessment Questionnaire Disability Index; ADL: activities of daily living.

Finally, we evaluated whether the demographic, anthropometric, and clinical data, pulmonary function variables, physical function variables, and chest CT findings could independently predict GA-T time (Table 3). Among the 14 variables that were candidates to enter the explanatory model for GA-T time (age, weight, height, body mass index, time since diagnosis, CDAI, CT involvement, FVC, DLco, FVC/DLco, maximal inspiratory pressure, maximal expiratory pressure, SIIIN_2_, and HAQ-DI), three were excluded because of high collinearity (weight, height, and the FVC/DLco ratio). On stepwise forward regression analysis, the SIIIN_2_ and HAQ-DI were the only variables that independently predicted the GA-T time, explaining 40% of its variability. Multicollinearity was not identified in this explanatory model as determined by a low VIF value (VIF=1.26). The nine variables that did not enter this model had P>0.10 and a maximum VIF of 2.98.

**Table 3:** Stepwise forward regression analysis for the phase III slope of the nitrogen single-breath washout using demographic, anthropometric and clinical data, lung function, physical function, and chest computed tomography findings.

Outcome variable	Independent variables	B	SEB	P value	Cumulative R^2^	VIF
Ln Glittre ADL-test time	SIIIN_2_	0.0007	0.0002	0.001	0.34	1.26
	HAQ-DI	0.187	0.096	0.039	0.40	1.26

Ln: natural logarithm; ADL: activities of daily living; B: regression coefficient; SEB: standard error of the regression coefficient; R^2^: determination coefficient; VIF: variance inflation factor; SIIIN_2_: phase III slope of the nitrogen single-breath washout; HAQ-DI: Health Assessment Questionnaire Disability Index.

To provide context for interpreting the null findings, a *post hoc* power analysis was performed using G*Power 3.1.1 software (https://www.psychologie.hhu.de/arbeitsgruppen/allgemeine-psychologie-und-arbeitspsychologie/gpower.html) based on the actual sample size (n=43) and the observed correlations between the main outcome (GA-T time) and the other studied variables. Based on *a priori* type-I error α=0.05 (two-tailed), a complete-case analysis showed that the observed significant effects were detected with a power ranging from 99 to 53%. For the regression model for GA-T time, the significant effect was observed with a power of 98%, showing the adequacy of the studied sample size to obtain significant results (33).

## Discussion

The lungs are frequently affected in RA and can play a crucial role both in the onset of autoimmunity and in the perpetuation of the disease (8). On the one hand, it is hypothesized that RA begins in the synovial tissue after an immune response against citrullinated proteins; on the other hand, it is believed that the collapse of immunological tolerance initially occurs in the lungs through an immune response against citrullinated proteins that secondarily spread to the joints (8). This interrelationship between the respiratory system and the musculoskeletal system is intriguing and raises questions about the contribution of ventilation inhomogeneity to the functional exercise capacity of individuals with RA. The main findings of the present study were that in women with RA, greater ventilation distribution heterogeneity corresponded to greater difficulty in performing the GA-T. The extent of the disease on CT was associated with GA-T time. In these patients, the worse the physical function was, the greater the difficulty of performing the GA-T. In addition, ventilation distribution heterogeneity and worse physical function explained 40% of the variability in GA-T time. To the best of our knowledge, this is the first study to evaluate the relationship between pulmonary function and GA-T multitasks in RA.

RA-PD can involve both the parenchyma and airways of various calibers. The involvement of the airways encompasses a number of abnormalities, including follicular bronchiolitis, bronchiectasis, and obliterative bronchiolitis; the latter is SAD characterized by the destruction of the bronchial epithelium and airflow obstruction (34). Taken together, these changes may result in ventilation distribution heterogeneity (7). Similar to a study by Bessa et al. (9), we observed that the increase in the SIIIN_2_, which is a marker of SAD and ventilation distribution heterogeneity, was the most common finding for pulmonary function and was observed in approximately 60% of patients. Because we excluded those who smoked >10 pack-years, we believe that these changes cannot be attributed to smoking. This is important because the pathogenesis of pulmonary changes in RA involves several cell types and a complex interaction between different cellular compartments, triggered by environmental factors associated with the disease (including smoking) and systemic inflammation, which results in consecutive damage to lung tissue and local inflammatory changes (8).

In the present study, we used the GA-T to assess functional exercise capacity because, in addition to incorporating upper and lower limb activities, this parameter reproduces daily activities involving multiple ADLs, including unsupported arm activities, standing up, walking, and climbing stairs (14). We observed that women with RA required more than twice the time expected to complete it when the data were compared to the values predicted for healthy Brazilian women (30). The median (interquartile range) GA-T time was also substantially higher than that observed in women with systemic sclerosis (SSc, 360 s *vs* 253 s) recently published in a study by Nonato et al. (29), suggesting that RA may have a more negative impact on the organ systems required to complete the GA-T activities than SSc. In fact, in addition to affecting the joints of the hands and wrists, arthritis in the feet is common in RA, and approximately 80% of individuals with RA report foot problems (16). Although our study is a pioneer in the use of the GA-T in RA patients, Cimen et al. (2) found that patients with RA presented lower values for the distance walked in the 6MWT than did the healthy controls; according to these authors, the shorter distance traveled in the 6MWT can be explained at least in part by inactivity during the active inflammatory period of the disease and by continuous inactivity during remission. In that study, the distance traveled in the 6MWT was not correlated with pulmonary function assessed by spirometry and respiratory muscle strength, which points to the need to evaluate other PFTs that may impact the reduced functional exercise capacity of RA patients.

Ventilation distribution heterogeneity reflects the efficiency of gas transport in both central airways (convection-dependent) and peripheral airways (diffusion-dependent) (23); therefore, its measurement may be more sensitive to lung damage in RA. In the present study, we observed that SIIIN_2_ showed a good correlation with GA-T time, and importantly, it was the only pulmonary function variable included in the multiple regression model to explain the changes in GA-T time. This finding suggested that SIIIN_2_ may be an interesting measure for the follow-up of individuals with RA; however, there is a need for longitudinal studies that can prove its usefulness in clinical practice. Despite not having entered our regression model, both DLco and the FVC/DLco ratio showed significant correlations with GA-T time. Although DLco is a sensitive marker for predicting the presence of RA-PD (34), the FVC/DLco ratio has been increasingly used in collagen diseases as a marker of vascular damage. In this sense, the importance of early identification of vascular damage in patients with RA has recently been emphasized, especially because there are several treatment options available; symptoms can be treated and right heart failure can be avoided (35). In our study, patients with extensive disease on CT required more time to perform the GA-T multitasks, which shows the close relationship between lung structural damage and poor performance during exercise.

In the present study, 40% of the variability in GA-T time was primarily explained by ventilation distribution heterogeneity, followed by worse physical function as assessed by the HAQ-DI. Unlike the study by Reis et al. (30), who developed Brazilian reference equations in healthy individuals, none of the demographic or anthropometric variables explained the variability of GA-T time in our study, indicating that the disease burden had a strong influence when measuring functional exercise capacity in people with RA. The HAQ-DI is a questionnaire that assesses the impact of disease on physical function and disability. Our findings are in line with those of Douglas-Withers et al. (3) who also evaluated patients with RA and observed a significant association between the HAQ-DI and the Timed Up and Go (TUG) test; interestingly, this test has certain similarities to the GA-T, such as rising from a chair and walking. However, unlike the GA-T, the TUG test does not incorporate upper limb activities, which are often the main reasons for disability in this population. Our results also support recommendations by the American College of Rheumatology that the HAQ-DI must be repeatedly evaluated in clinical practice to track patient outcomes and guide shared decision-making (36). However, it is important to highlight the limitations of the HAQ-DI in the evaluation of more complex daily activities and its lack of sensitivity regarding the detection of changes in patients with low weakness (“floor effect”) (37).

The strength of our study is that it was the first to evaluate the contribution of ventilation distribution heterogeneity to functional capacity during exercise assessed while performing multiple tasks that simulate ADLs (GA-T). Despite the interesting results, the present study has limitations. First, generalization of our results may be limited by our small sample; however, RA has a low prevalence, and the study design included several evaluation tools. Second, we evaluated only women with RA; however, most RA patients are women, with a female to male ratio of 3:1 (38). Third, we did not evaluate a control group, although the results were compared to recently published results for healthy Brazilian individuals (30). Fourth, the cross-sectional design of this study limited our ability to establish causal relationships between variables. Fifth, we did not evaluate radiological deformities of the hand and foot joints, which may be associated with strength measurements and thus impact functional exercise capacity.

Controlled longitudinal studies are needed to determine the clinical applicability of the test in this population. Because hand deformities can strongly impact functional exercise capacity in RA, we think that determining the time to complete the shelf task can be interesting in these individuals as observed in patients with SSc (27). Despite these limitations, notably, the GA-T is an easy test to administer and provides useful information on the functional status of patients; thus, the analysis of the feasibility of administering the GA-T in a clinical setting would be a natural extension of the present study. Furthermore, ventilation distribution heterogeneity measured using the N_2_SBW test may be interesting in the follow-up of RA cases because this variable is a measure of lung function that can be more strongly affected by pulmonary involvement in RA.

In conclusion, the present study showed that in women with RA and varying extents of disease on chest CT, relationships existed between GA-T time and ventilation distribution, pulmonary diffusion, and physical capacity. Moreover, ventilation distribution heterogeneity and worse physical function substantially explained the variability in GA-T time.
